# Environmental and ecological factors mediate taxonomic composition and body size of polyplacophoran assemblages along the Peruvian Province

**DOI:** 10.1038/s41598-019-52395-z

**Published:** 2019-11-04

**Authors:** Christian M. Ibáñez, Melany Waldisperg, Felipe I. Torres, Sergio A. Carrasco, Javier Sellanes, M. Cecilia Pardo-Gandarillas, Julia D. Sigwart

**Affiliations:** 1 Departamento de Ecología y Biodiversidad, Facultad de Ciencias de la Vida, Universidad Andres Bello, Santiago, Chile; 2 Departamento de Ecología, Facultad de Ciencias Biológicas, Pontificia Universidad Católica de Chile, Santiago, Chile; 3 Departamento de Ciencias Ecológicas, Facultad de Ciencias, Universidad de Chile, Santiago, Chile; 4 Departamento de Biología Marina, Facultad de Ciencias del Mar, Universidad Católica del Norte, Larrondo 1281, Coquimbo, Chile; 5 Millennium Nucleus for Ecology and Sustainable Management of Oceanic Islands (ESMOI), Coquimbo, Chile; 6 Marine Laboratory, Queen’s University Belfast, Portaferry, N. Ireland

**Keywords:** Biogeography, Community ecology

## Abstract

Intertidal communities’ composition and diversity usually exhibit strong changes in relation to environmental gradients at different biogeographical scales. This study represents the first comprehensive diversity and composition description of polyplacophoran assemblages along the Peruvian Province (SE Pacific, 12°S–39°S), as a model system for ecological latitudinal gradients. A total of 4,775 chitons from 21 species were collected on twelve localities along the Peruvian Province. This sampling allowed us to quantitatively estimate the relative abundance of the species in this assemblage, and to test whether chitons conform to elementary predictions of major biogeographic patterns such as a latitudinal diversity gradient. We found that the species composition supported the division of the province into three ecoregional faunal groups (i.e. Humboldtian, Central Chile, and Araucanian). Though chiton diversity did not follow a clear latitudinal gradient, changes in species composition were dominated by smaller scale variability in salinity and temperature. Body size significantly differed by ecoregions and species, indicating latitudinal size-structure assamblages. In some localities body size ratios differed from a random assemblage, evidencing competition at local scale. Changes in composition between ecoregions influence body size structure, and their overlapping produce vertical size segregation, suggesting that competition coupled with environmental conditions structure these assemblages.

## Introduction

Environmental factors (e.g. temperature, productivity) are the main drivers modulating latitudinal gradients of species richness at different spatial and temporal biogeographical scales^1^. Different ecological mechanisms have been suggested to underpin these gradients in species richness. One of them is the “competitive exclusion hypothesis”, which predicts a positive relationship between primary productivity and species diversity, and may directly relate factors such as lower productivity, stress, and lack of resources, to the limited number of species that can survive in a specific environment^2–4^. Therefore, when productivity increases, species richness also increases until a point where it becomes regulated by intense competition, which can cause an important competitive exclusion^2,3^. Another possible explanation is the “species-energy hypothesis”, which suggests that species with small populations are highly vulnerable to stochastic events; hence, species persistence over time would depend on their ability to maintain a stable minimum population^1,5,6^. An environment with higher productivity will be able to support more species with viable minimum populations^7^. One way to infer competition within communities is to evaluate the body size ratio between species, according to Hutchinson’s hypothesis, which states that a minimum space between species is necessary for coexistence^8^. This approach has produced a huge number of ecological studies searching for body size patterns of coexisting species^9–11^. Most controversy over statistical analyses of size overlap data (based on random assemblages)^9,10^ has been solved based on null models^11^.

The Southeastern Pacific (SEP) is composed of three biogeographic provinces: Panamian, Peruvian and Magellan^12^. The Peruvian Province, which extends from the Gulf of Guayaquil at 3°S to 42°S on the Chilean coast, is further subdivided in four recognised ecoregions (Central Peru, Humboldtian, Central Chile and Araucanian), where the marine species composition and richness is determined by oceanographic (e.g. temperature, productivity) and topographic features (e.g. coastal shelf area)^13–18^. One of the fundamental tenets of biogeography is the latitudinal diversity gradient: there are more species in the tropics^19^. Yet, a striking absence of clear latitudinal diversity patterns in the SEP have been found in different assamblages at the community level^20–24^, suggesting that the factors determining the distribution of organisms could be more prominently influenced by other factors, including both natural and anthropogenic drivers that locally modify oceanographic traits (e.g. upwelling zones, Ekman transport, and biogeographic transitions around 30°S) (see^20,21^). This geographic variation in local richness of intertidal and subtidal communities along SEP exhibit a complex pattern, where areas of high and low species richness appear to be interspersed haphazardly^21–23^.

Much of the global data on biogeographic patterns comes from the study of molluscs, because of their ubiquity and deep fossil record^25^. Molluscs are also the most studied marine taxa in relation to gradients of species richness in the SEP; however, a large density of samples is required to test biogeographic hypotheses^14,16,17,26,27^. Within this group, the polyplacophorans, or chitons, stand out as important grazers in rocky intertidal habitats, capable of affecting the community structure due to their locally high abundances^28–33^. Chitons are exclusively marine animals, and many species are stenohaline^34,35^, tolerating very low levels of salinity (e.g. *Plaxiphora aurata* in Chilean fjords)^35,36^. Therefore, it is possible that diversity and abundance of these species could be strongly influenced by local scale environmental factors, such as temperature and/or salinity. Another interesting pattern is the vertical segregation in size structure in intertidal habitats, where small chiton species and small individuals of larger species are found at higher habitats, whereas larger species occur at lower levels in rocky shores^37^.

The present study aims to describe and quantify for the first time the diversity (richness and abundance) and composition of polyplacophoran asemblages along 2,500 km of coast in the Peruvian Province to: 1) compare their faunistic similarities/differences among ecoregions, 2) evaluate the associations between diversity and environmental variables, and 3) evaluate potential size segregation by inter-specific competition through comparisons with null models of body size ratios. Considering the above, we hypothesize that environmental factors, and the effect of spatial competition, could determine the body size and distribution limits of chitons along the coasts of the South-eastern Pacific Ocean, generating a latitudinal gradient in species composition and diversity.

## Results

**Figure 1:**
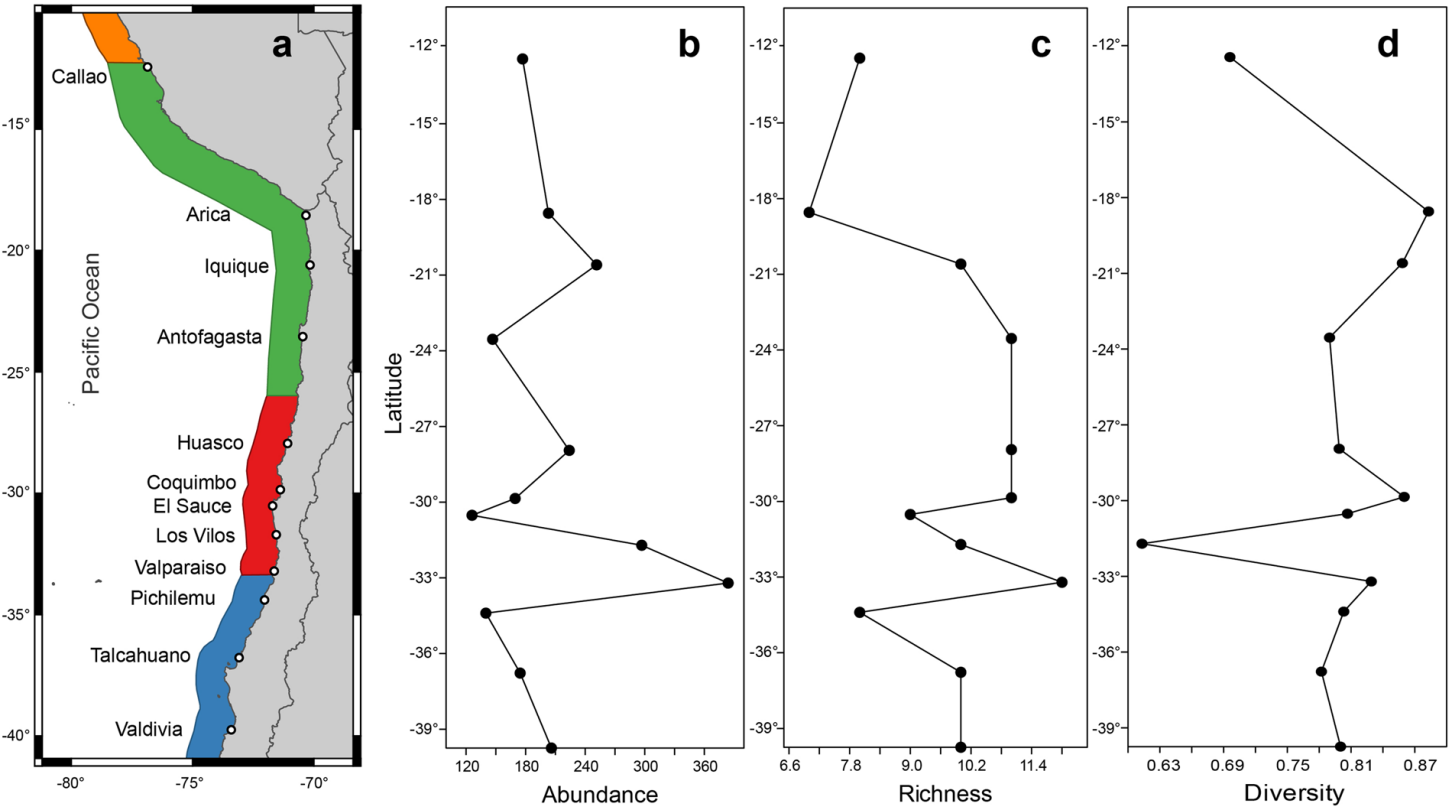
Sampling sites of polyplacophoran assemblages along 2,500 km of coast line in the Peruvian Province, from Callao, Peru (12°S) to Valdivia, Chile (39°S); (**a)** Map showing sampling sites and marine ecoregions represented by color areas in the coast-line (orange: Central Peru; green = Humboldtian; red = Central Chile; blue = Araucanian) (**b)** Relative abundance, (**c**) Species richness, and (**d**) Diversity.

**Figure 2:**
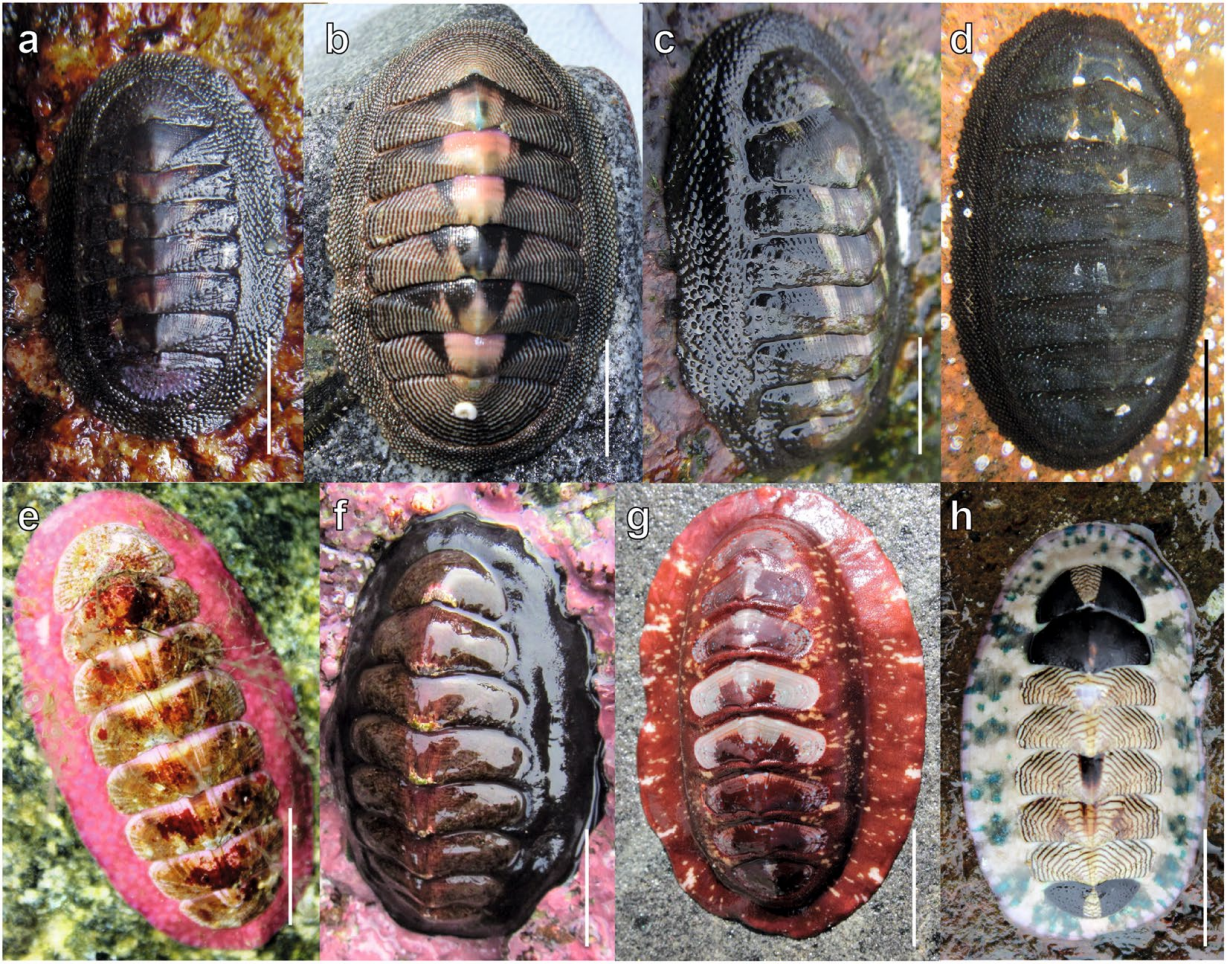
Chitons of the family Chitonidae found in the Peruvian Province; (**a**) *Chiton barnesii* (Coquimbo, scale bar = 20 mm). (**b**) C. *cumingsii* (Coquimbo, scale bar = 10 mm). (**c**) *C. granosus* (Valparaiso, scale bar = 10 mm.) **(d**) *C. magnificus* (Valparaiso, scale bar = 20 mm). (**e**) *Tonicia calbucencis* (Valdivia, scale bar = 5 mm). **(f**) *T. chilensis* (Valparaiso, scale bar = 15 mm). (**g**) *T. disjuncta* (Los Vilos, scale bar = 20 mm). (**h**) *T. fremblyana* (Callao, scale bar = 10 mm). Photographs credits: C.M. Ibáñez and M.C. Pardo-Gandarillas.

**Figure 3:**
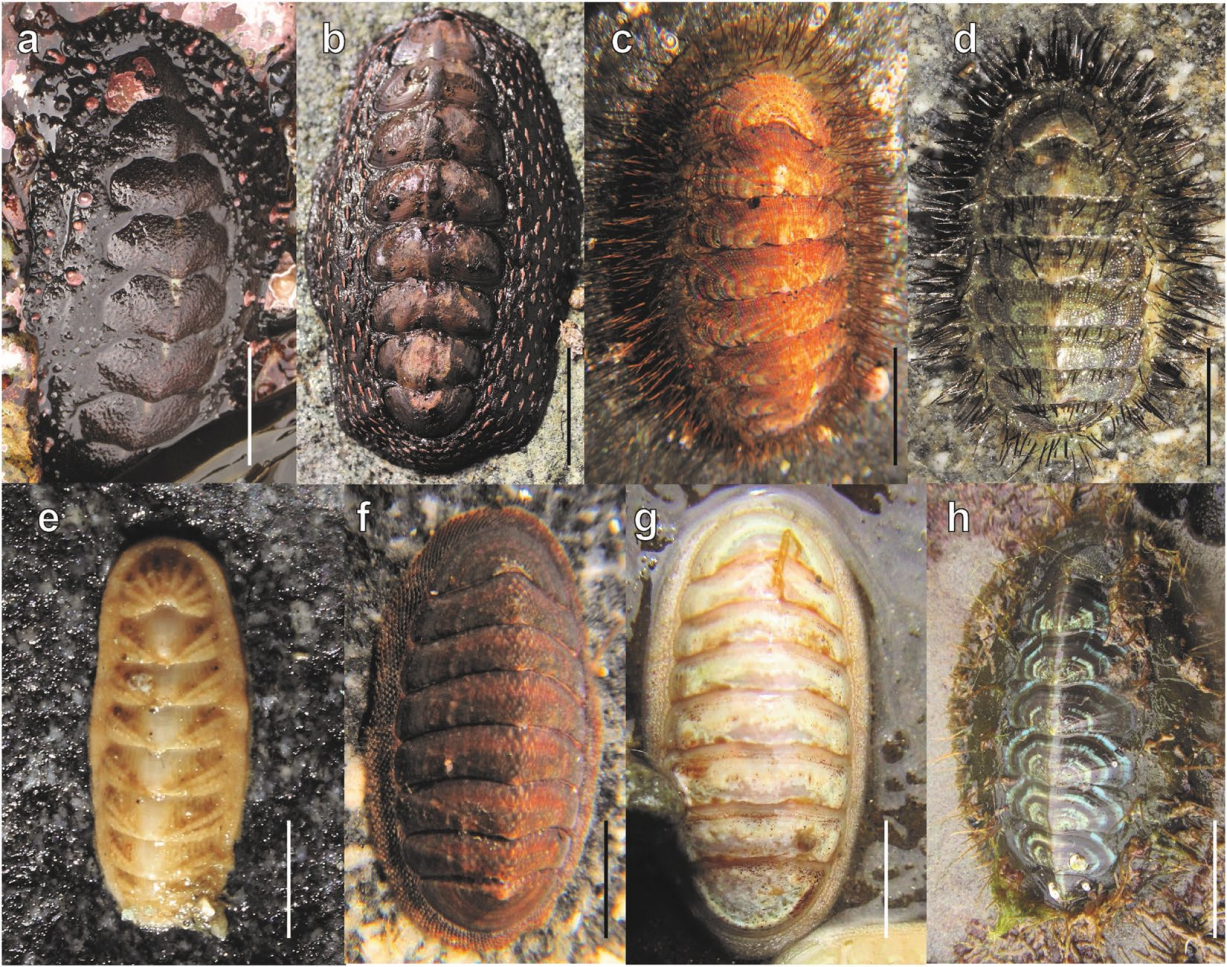
Chitons of the family Chitonidae, Chaetopleuridae, Callistoplacidae, Ischnochitonidae and Mopaliidae found in the Peruvian Province; (**a**) *Acanthopleura echinata* (Antofagasta, scale bar = 30 mm), (**b**) *Enoplochiton niger* (Coquimbo, scale bar = 20 mm), **(c**) *Chaetopleura benaventei* (Talcahuano, scale bar = 10 mm), (**d**) *C. peruviana* (Coquimbo, scale bar = 10 mm), (**e**) *Calloplax vivipara* (Coquimbo, scale bar = 5 mm), (**f**) *Ischnochiton pusio* (Valparaiso, scale bar = 5 mm), (**g**) *I. stramineus* (El Sauce, scale bar = 5 mm), (**h**) *Plaxiphora aurata* (Valdivia, scale bar = 10 mm). Photographs credits: C.M. Ibáñez and M.C. Pardo-Gandarillas.

A total of 4,775 chitons belonging to 21 species were collected on 12 localities along the Peruvian Province (Fig. 1a). The most abundant species corresponded to *Chiton cumingsii* (relative abundance, RA = 64 chitons/hours/persons), *C. granosus* (RA = 26), *Tonicia fremblyana* (RA = 65), *T. calbucensis* (RA = 24), *C. magnificus* (RA = 17) (Fig. 2b–e,h, respectively), *Acanthopleura echinata* (RA = 7.5) and *Enoplochiton niger* (RA = 9.5) (Fig. 3a,b). Along the Peruvian Province, the highest abundances (RA = 177) were observed in Callao (Peru), whereas the lowest (RA = 32) were observed in El Sauce (Chile) (Table 1, Fig. 1b).

**Table 1:** Relative abundance (RA = chitons/hour/person) and diversity variables (S, J) of the polyplacophoran species collected in 12 localities along the Peruvian Province (12°S–39°S).

Species/Ecoregion	Callao	Arica	Iquique	Antofagasta	Huasco	Coquimbo	El Sauce	Los Vilos	Valparaiso	Pichilemu	Talcahuano	Valdivia
	Humboldtian				Central Chile					Araucanian		
*Acanthopleura echinata*	7.5	3	6.9	3	4	2.6		5.5	5.3	7.4	1.8	
*Callistochiton pulchellus*			7.9									
*Calloplax vivipara*			0.3			0.3	1.9	0.8	2.1			
*Chaetopleura benaventei*											0.9	2.8
*Chaetopleura hennahi*	1.5											
*Chaetopleura peruviana*	1	0.5	1.5	0.3	2.4	3.8	5.1	0.6	4.8	0.5	0.6	3.3
*Chiton barnesii*				0.4	2.7	2.9	12	0.3				0.3
*Chiton cumingsii*	64	12	13	1.8	24	14	4.3	42	18	0.6	8.8	1.3
*Chiton granosus*	26	14	14	6.3	3.7	2.5	0.5	4.5	12	12	9.5	13
*Chiton magnificus*					4.1	7.4	2.4	1.1	17	5.8	13	8.5
*Enoplochiton niger*	9.5	9	1.5	5	6.6	3.3						
*Gallardoia valdiviensis*												0.3
*Ischnochiton punctulatissimus*			6	0.1								
*Ischnochiton pusio*				3.6	4.4	2.6	0.3	0.6	3.6			
*Ischnochiton stramineus*							1.1					
*Plaxiphora aurata*				0.2					0.5		0.3	1
*Tonicia calbucensis*		4.5	2	1	3.4	2.8	3.8	9.4	24	2.8	5.3	13
*Tonicia chilensis*					0.3				5.3	5.3	4	9.3
*Tonicia disjuncta*								0.8	0.3		0.1	
*Tonicia fremblyana*	65	7.8	1.1	6	0.8	0.9			3.4	0.5		
*Tonicia swainsoni*	3											
**Diversity indices**												
Abundance (RA)	177	51	63	37	56	42	32	74	96	35	44	52
Richness (S)	8	7	10	11	11	11	9	10	12	8	10	10
Evenness (J)	0.70	0.88	0.86	0.79	0.80	0.86	0.81	0.61	0.83	0.80	0.78	0.80

The rarefaction curves obtained for each locality showed a saturation around 50 RA, reached between 6 to 10 species (Fig. S1); therefore, the estimated richness based on the RA matrix was strong enough to represent the diversity of chitons (Fig. S1).

Chiton assemblages from the Peruvian Province had a comparatively high (and similar) diversity among localities, with around 7–12 species per site and higher local evenness rather than dominance of a single species (Table 1). In fact, in each ecoregion more than 60% of species are rare (<10 RA, Table 1). The assemblage of chitons found along the Peruvian Province evidenced the highest richness in Valparaiso (12 species), followed by Antofagasta, Huasco, Coquimbo (11 species), Iquique, Los Vilos, Talcahuano, Valdivia (10 species), El Sauce (9 species), Callao, Pichilemu (8 species) and finally Arica (7 species) (Table 1; Fig. 1c). The highest and lowest evenness (J) were evidenced in Arica and Los Vilos, respectively (Table 1).

**Figure 4:**
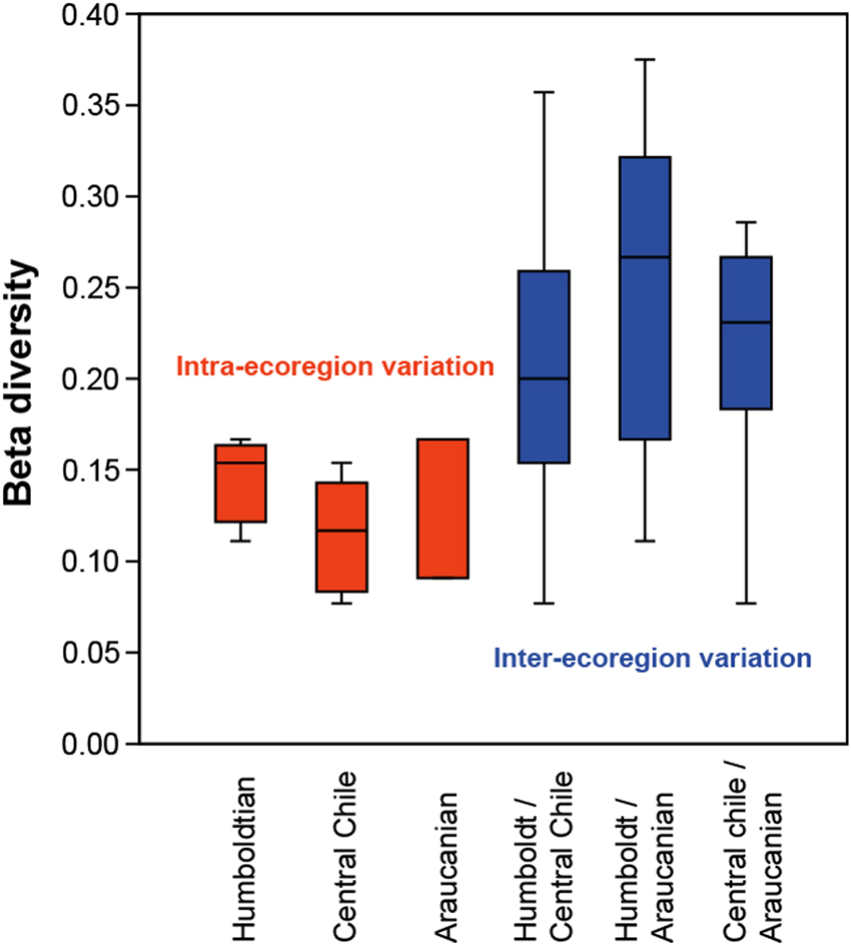
Beta-diversity variation at intra (red plots) and inter-ecoregions (blue plots) levels along the Peruvian Province.

**Figure 5:**
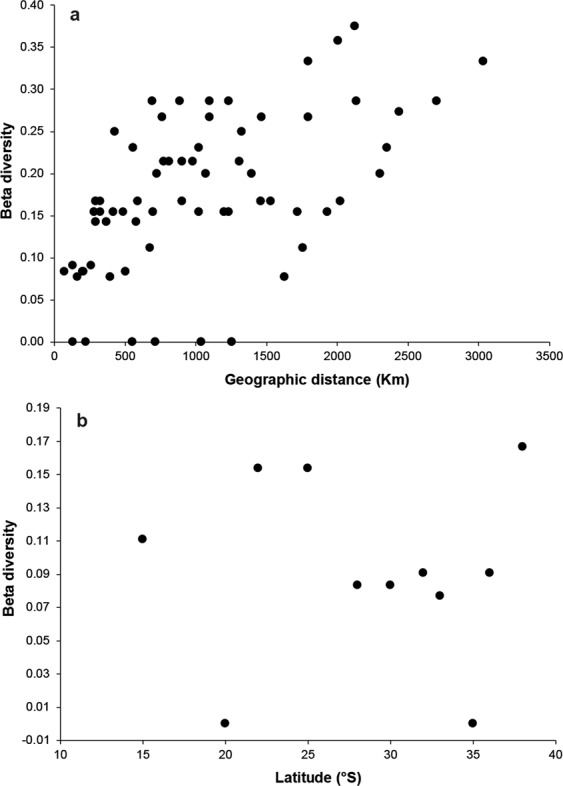
Relationship between beta-diversity and; (**a**) geographic distance (kms), and (**b**) latitude (°S) among sampling localities along the Peruvian Province.

Beta diversity was relatively low at the intra-ecoregion level (0.07–0.16) but higher among ecoregions (0.28–0.37; Fig. 4), suggesting a species replacement pattern related to distance (km) among sites (Fig. 5a). For example, *Callistochiton pulchellus* and *Chaetopleura hennahi*, *Ischnochiton punctulatissimus* and *Tonicia swainsoni* are unique in the Humboldtian ecoregion, while others were exclusive to the Araucanian ecoregion (e.g. *Chaetopleura benaventei* and *Gallardoia valdiviensis*). Despite positive value of the Mantel correlation coefficient (*r* = 0.53, *P* < 0.001), the beta diversity did not evidence significant or consistent correlation to latitude (*r* = −0.017, *P* = 0.958, Fig. 5b).

**Figure 6:**
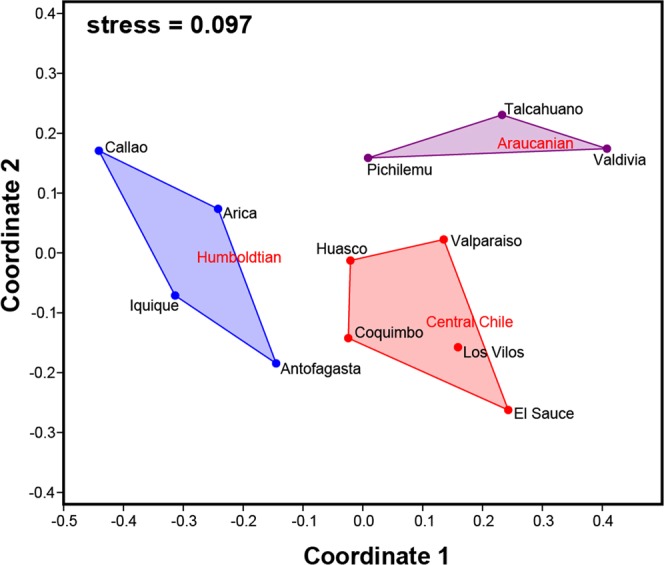
Non-metric Multidimensinal Scaling (nMDS) of chiton assemblages from the Peruvian Province using Jaccard similarity.

The species composition (Jaccard) carried out by the nMDS analysis, supported the existence of three faunal assemblages related to the different ecoregions (Fig. 6). A northern group between 12°S–24°S corresponding to the Humboldtian ecoregion (i.e. Callao, Arica, Iquique and Antofagasta; see Fig. 6). A second group between 27°S -34°S corresponding to the Central Chile ecoregion (i.e. Huasco, Coquimbo, El Sauce, Los Vilos and Valparaiso; Fig. 6). A third group between 34°S–40°S that conformed the Araucanian ecoregion (i.e. Pichilemu, Talcahuano and Valdivia; Fig. 6). Significant differences in species composition between ecoregions were found (PERMANOVA Jaccard, *F*_(2,9)_ = 5.104, *P* < 0.001), with SIMPER analysis evidencing the highest relative contributions of *Chiton cumingsii*, *C. magnificus* and *C. granosus*, the only three species with average relative abundances >10% (Online Appendix Table S1, Fig. 2b–d, respectively). None of the diversity variables (species richness, relative abundance, and evenness) showed significant differences between ecoregions (PERMANOVA, 0.108 < *F* < 1.607; *P* > 0.05).

Neither the ordinary least square regression analysis (OLS) nor the spatial autoregressive model (SAR) evidenced significant relationships between abundance, richness and eveness with the environmental variables evaluated (Table 2), due to a high spatial autocorrelation (ρ = 0.969). Only species composition (Jaccard) was significant related with temperature and salinity (Table 2).

**Table 2:** Summary of regression analyses; Ordinary Least Square Regression (OLS) and Spatial Autoregressive Model (SAR) for the relationships between diversity (12°S–36.8°S) and Chlorophyll-a, Temperature, and Salinity.

	OLS			SAR		
	b	r^2^	P-value	b	r^2^	P-value
**Evenness**						
Chlorophyll-a	0.003	0.013	0.720	0.008	0.303	0.509
Temperature	0.007	0.179	0.576	0.004	0.100	0.807
Salinity	0.014	0.015	0.975	−0.004	0.303	0.938
**Richness**						
Chlorophyll-a	−0.190	0.163	0.636	−0.108	0.476	0.245
Temperature	−0.169	0.053	0.954	−0.018	0.517	0.747
Salinity	−0.585	0.064	0.414	−0.695	0.628	0.436
**Relative abundance (RA)**						
Chlorophyll-a	−5.396	0.053	0.650	−6.104	0.167	0.475
Temperature	−1.361	0.001	0.691	7.247	0.171	0.975
Salinity	−2.482	<0.001	0.992	0.493	0.170	0.975
**Composition (Jaccard)**						
Chlorophyll-a	−0.026	0.149	0.385	−0.024	0.647	0.084
**Temperature**	−0.096	0.827	<0.001	−0.104	0.933	<0.001
**Salinity**	−0.309	0.857	<0.001	−0.301	0.851	<0.001

^*^Significant p-values < 0.05.

**Figure 7:**
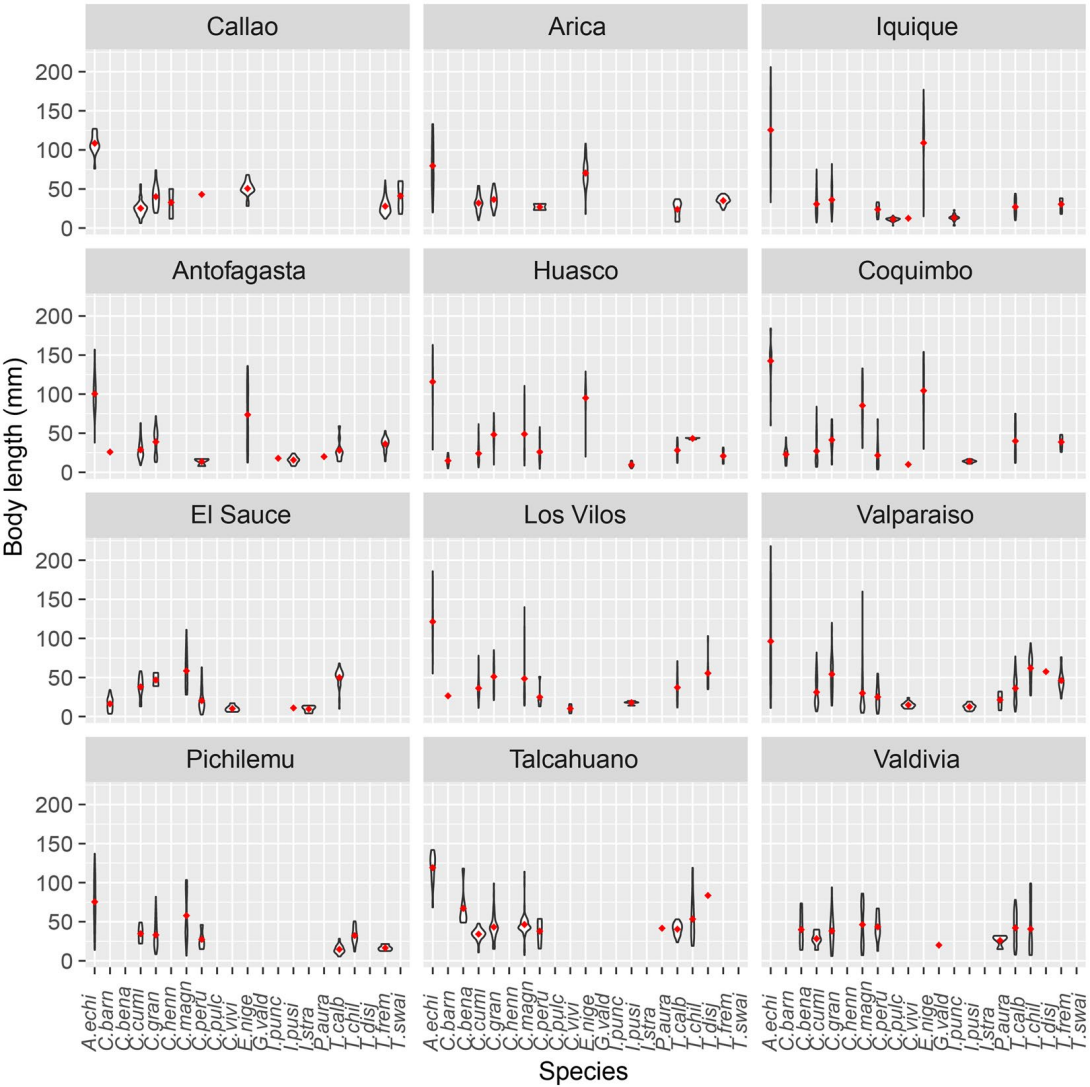
Violin plots by locality and species of chitons body length (mm) from the Peruvian Province. Abbreviations: *A. echi*: *Acanthopleura echinata, C. barn*: *Chiton barnesii, C. bena*: *C. benaventei, C. cumi*: *C. cumingsii, C. gran*: *C. granosus, C. magn*: *C. magnificus, C. peru*: *Chaetopleura peruviana, C. henn*: *C. hennahi, C. vivi*: *Calloplax vivipara, E. nige*: *Enoplochiton niger, G. vald*: *Gallardoia valdiviensis, I. punc*: *Ischnochiton punctulatissimus, I. pusi*: *I. pusio, I. stra*: *I. stramineus, P. aura*: *Plaxiphora aurata, T. calb*: *Tonicia calbucencis, T. chil*: *T. chilensis, T. disj*: *T. disjuncta, T., frem*: *T. fremblyana, T. swai*: *swainsoni*.

Overall, most chitons were small (18 species; <50 mm TL), whith only three species (*Acanthopleura echinata*, *Chiton magnificus* and *Enoplochiton niger*; see Online Appendix Table S2, Fig. 7) corresponding to large chitons (>50 mm TL). Significant differences in chiton body lengths were found to be structured by species and ecoregions, with significant interactions between most factors (Table 3). The year was the only factor without significant differences (Table 3). However, species was the only factor that explained a high proportion of deviance (56%; see Table 3). Mean minimum segment lengths in each locality were heterogeneous (59.1–88.8 mm), being higher at northern locations and decreasing through the south (Table S3). Maximum chitons body length at each locality was correlated with mean minimum segment length (*r* = 0.59, *P* < 0.05), suggesting that in places with larger chitons they are highly segregated by size. Body size ratios were highly variable (0.0–4.6) and showed significant differences from a random assemblage that was not structured by competition only in some localities, where we suggest some evidence of competition by size overlap at local scale (Table S3).

**Table 3:** Summary of GLM analyses for the relationships between body size and ecoregions, year, and species.

	D.F.	Deviance	F	P	%Deviance
NULL	4724	4859186			
Ecoregion	2	2747	3.236	0.0394	0.06
Year	1	92	0.217	0.6409	0.002
Species	19	2722337	337.630	<0.05	56.02
Ecoregion:Year	2	16832	19.831	<0.05	0.35
Ecoregion:Species	19	88788	11.011	<0.05	1.83
Year:Species	16	33757	4.971	<0.05	0.69
Ecoregion:Year:Species	14	20878	3.514	<0.05	0.43

## Discussion

Our results showed an absence of a latitudinal gradient in abundance and diversity of coastal polyplacophorans in the Peruvian Province, supporting prior results on this coastline^21–24^. However, the size structure abruptly changed along the coast as a consequence of changes in the latitudinal taxonomic composition. These findings agree with previous descriptions^37–39^ and suggest that species assemblages do conform to the biogeographic province they inhabit, similar to what has been described in many other SE Pacific marine invertebrate species such as crustaceans, molluscs and echinoderms^12,15,16^.

The Peruvian Province has a diversity of chitons similar to that observed in rocky shore ecosystems around the Southeastern Pacific, Baja California and Costa Rica, where up to 12 species of chitons have been reported per site^35,40,41^. In the other hand, the lower diversity observed in tropical areas such as Ecuador and northern Peru, would be associated with the low diversity of seaweeds and the scarce rocky ecosystems^42^. It is well known that chitons prefer hard substrata on rocky shores, where some species may be found in high abundances^29,37^.

As shown here, nearby localities tended to have similar species, with composition varying significantly as the distance between localities increased. This same pattern has been observed in subtidal and intertidal fishes^42^ as well as in benthic communities^21–23,43–46^. In this context, Beta diversity (in addition to have the component of species replacement) can also be evaluated from the perspective of similarity between habitats or species’ nestedness^47,48^. In the case of the SEP chitons, replacement did not show a latitudinal pattern since these species rarely have small geographical ranges, and dissimilarity between close localities remained low through the latitudinal gradient. The highest species replacement occurred towards the northern part of Central Chile ecoregion (Huasco and Coquimbo) and the southern end of the Peruvian Province (Valdivia), being evidenced as a direct relationship between geographic distance and Beta diversity.

The taxonomic composition variation of chiton assemblages along the sampled localities was reflected in the faunistic groups supported by the nMDS analysis. Variation in the assemblages’ composition was associated to temperature and salinity gradient along the Peruvian Province. Also, the concordance of three faunal groups with some biogeographic breaks was evident, similar to what has been documented in other coastal invertebrates^27^. Some chiton species showed strong endemism, being unique in the Humboldtian ecoregion (e.g. *Callistochiton pulchellus*, *Chaetopleura hennahi*, *Ischnochiton punctulatissimus*, *Tonicia swainsoni*), while others were exclusive to the Araucanian ecoregion (e.g. *Chaetopleura benaventei*, *Gallardoia valdiviensis*). Those species strongly contributed to the differences in species composition. Contrary to such patterns, some chiton species also inhabited different ecoregions (e.g. *Acanthoplerura echinata*, *Chiton granosus*, *C. cumingsii*, *T. calbucensis* and *Chaetopleura peruviana*) maintaining the low levels of replacement observed among localities.

For most intertidal invertebrates along the SEP, sea surface temperature and salinity have been shown to play important roles^15,20,21,49^, contrasting to our findings where no significant correlations with overall chiton diversity where observed. The Humboldt ecosystem is characterized by coastal upwelling in Peru and Chile, and is tightly related to the spatial and temporal dynamics of coastal environments^15,50^. In Chile, two main regions of coastal upwelling have been detected, one in the Mejillones Peninsula (northern Antofagasta; ~23°S) and the other between Punta Lengua de Vaca and Punta Pájaros (southern Coquimbo; ~30°S)^15,50^. Accordingly, these sites evidenced the higher richness and diversity of chitons found between Antofagasta and Valparaiso, localities exhibiting maximum levels of upwelling and productivity. However, chitons diversity was not correlated with chlorophyll-a (an indicator of upwelling events in the HCS) but would be more related to the local availability of favorable habitats (i.e. rocky shores) and food^42^, rather than to environmental variables. Along the Humboldt Current System, the sandy beaches are also frequent^15^, representing unsuitable habitat for chitons. These heterogeneous stretches of beaches, combined with rocky shores, could explain the heterogeneous pattern and the absence of a cline of diversity of chitons found in this study. In fact, it has been suggested that local and small-scale processes could be determinants of the diversity and composition of intertidal communities in Chile, at least between Arica and Valparaíso^23^.

Chiton body lengths showed a high spatial variability and a latitudinal cline in assamblage structure. In northern localities of the Humboldtian and Central Chile ecoregions, assamblages were composed by large and small species, but in southern localities of the Araucanian ecoregion assamblages were composed only by medium and small species. These changes in body size structure were related to changes in species composition. The largest species (>100 mm; *E. niger* and *A. echinata*, Table S2) inhabit from Coquimbo and Talcahuano through northern localities, respectively^39^. Intertidal chitons are generally thought to be larger than subtidal and deep-sea species^51^, and Southeastren Pacific chitons larger than closely-related species from other ecosystems elsewere, excepting for North Pacific^52^. The evolutionary colonization of the intertidal by chitons increased the competition among them and among other movile invertebrates such as limpets, needing bigger sizes to competitively exclude interspecifics^52,53^. In central Chile, the largest chitons inhabit zones of high energy on the lower intertidal, while small and juveniles coexist in protected zones in high intertidal zones^37^. This pattern suggests certain kind of competition or niche partitioning among chitons and among other invertebrates, since species of similar body size might not be able to coexist because they overlap too much in the use of shared resources^11^. However, it should be noted that different species of chitons, are known to have different diets and niches. For example, there is evidence to suggest that the radiation of large bodied *Mopalia* species in the NE Pacific was driven by niche partitioning to avoid direct competition^54^. Our results of body size ratio and minimum segment length overlap agreed with this hypothesis, since were different from body size simulated data in absence of competition. Another evidence of competition among chitons comes from diet, as several Chilean chitons overlap their diet resources among species and between other intertidal grazers, suggesting a strong competition (e.g. *Fissurella* spp. and *Scurria* spp.^33^).

Overall, this work represents the first faunistic and ecological description of polyplacophoran assemblages inhabiting along the Humboldt Current System, providing critical information for an adequate characterization of biodiversity, conservation and potential management strategies in this area, as 3 of the 21 species recorded correspond to artisanally-exploited resources in Chile and Peru.

## Methods

Polyplacophoran samplings were carried out in 12 localities during 2013–2016, between Callao (Peru; 12°S) and Valdivia (Chile; 39°S) (Fig. 1a), encompassing more than 2,500 km of coastline including three of the four previously mentioned ecoregions of the Peruvian Province (i.e. Humboldtian, Central Chile and Araucanian). Chitons were collected during low tide for a period of one to three hours, sampling on average 4 days per locality. Intertidal species were sampled by 1 to 3 people, while subtidal species by 1 to 2 divers through snorkelling at depths up to 5 m. The collected species were relaxed with 5% ethanol with sea water, flattened and preserved in 96% ethanol for further genetic analyses. Once in the laboratory, the total length (mm, including the girdle) of each chiton was measured to perform comparissons among species, ecoregions and years. In order to standardize the sampling effort, a relative abundance (RA) index was estimated, corrected by sampling time and number of people (RA = n°chitons/hour/person). Species identification was performed visually under stereomicroscopy using descriptions and taxonomic keys^55–58^.

To determine whether the RA matrix of species richness is representative of local diversity, a rarefaction curve^59^ was calculated for each locality. The expected number of species was estimated using the Chao-1 algorithm^59^, based on relative abundance.

In each locality, species richness (S) and evenness (J) were estimated using the RA data. To evaluate the replacement degree in species composition between localities and ecoregions, beta diversity was estimated using the Williams index^60^ defined as changes species composition with distance^61^. Correlation between geographical distance (km) and beta diversity was determined using the Mantel test with 5,000 permutations^62^.

Similarity between chiton assemblages was evaluated using a non-metric Multidimensinal Scaling (nMDS)^63^ performed with presence-absence data through Jaccard similarities. Differences in diversity variables and species composition between ecoregions were evaluated with one-way PERMANOVA using 10,000 permutations^64^. Subsequently, to determine which species differed significantly between-groups, a Percentage Similarity Analysis (SIMPER) was used^63^. Environmental variables such as chlorophyll-a (as a proxy of productivity [mg/m^3^]), sea surface temperature [SST, °C] and salinity [PSU] were used to test their relationship with different diversity variables and composition using ordinary linear regressions (OLS) and spatial autoregressive models (SAR), both performed in the software SAM v.4.0^51^. These environmental data (monthly averages 2000–2014) were obtained from the Bio-Oracle v2.0 database (http://www.oracle.ugent.be/) through the DIVA-GIS v.7.5 software^65^. Rarefaction curves, Diversity, Mantel test, nMDS, SIMPER and PERMANOVA analyses were performed in PAST v.3.22 statistical package^66^.

Chiton body length structure was compared between species, ecoregions and years using Generalized Linear Model (GLM) implemented in the statistical software R v 3.6.1^67^ In order to explore the possibility of competition, we estimated body size overlap between species in each locality, we generated 5,000 random matrices to create a random size assemblage in the software ECOSIM v7.72^68^. As size overlap metric, we used the minimum segment length (calculated as the difference in body sizes of two adjacent species) and absolute size differences for each locality. The minimum segment length corresponded to Hutchinson’s hypothesis, which stated that a minimum spacing between species is necessary for coexistence. The standardized effect size (SES) of the body size ratio, was calculated as: observed index – mean (simmulated indices)/standard deviation (simulated indices). If SES was greater than 2 or less than -2, it was considered as statistically significant with a probability <0.05^68,69^.

## Supplementary information

Supplementary

## References

[1] Willig MR, Kaufman DM, Stevens RD (2003). Latitudinal gradients of biodiversity: patterns, process, scale, and synthesis. Annu. Rev. Ecol. Evol. Syst..

[2] Grime JP (1973). Competitive exclusion in herbaceous vegetation. Nature.

[3] Grime, J. P. *Plant strategies and vegetation processes*. (John Wiley and Sons, 1979).

[4] Huston MA (1979). A general hypothesis of species diversity. Am. Nat..

[5] Coleman BD, Mares MA, Willig MR, Hsieh YH (1982). Randomness, area, and species richness. Ecology.

[6] Rosenzweig, M. L. *Species diversity in space and time*. (Cambridge University Press, 1995).

[7] Wright DH (1983). Species–energy theory: an extension of species-area theory. Oikos.

[8] Hutchinson GE (1959). Homage to Santa Rosalia or why are there so many kinds of animals?. Am. Nat..

[9] Simberloff D, Boecklen W (1981). Santa Rosalia reconsidered: size ratios and competition. Evolution.

[10] Wiens JA (1982). On size ratios and sequences in ecological communities: Are there no rules?. Annales Zoologici. Fennici..

[11] Gotelli, N. J., & Graves, G. R. Null models in ecology. *Smithsonian Institution* (1996).

[12] Camus PA (2001). Biogeografía marina de Chile continental. Rev. Chil. Hist. Nat..

[13] Astorga A, Fernández M, Boschi EE, Lagos N (2003). Two oceans, two taxa and one model of development: latitudinal diversity patterns of South American crabs and test for possible causal processes. Ecol. Lett..

[14] Valdovinos C, Navarrete SA, Marquet PA (2003). Mollusk species diversity in the Southeastern Pacific: why are there more species towards the pole?. Ecography.

[15] Thiel M (2007). The Humboldt Current System of northern and central Chile. Oceanographic processes, ecological interactions and socioeconomic feedback. Ocean. Mar. Biol. Annu. Rev..

[16] Ibáñez CM, Camus PA, Rocha FJ (2009). Diversity and distribution of cephalopod species off the coast of Chile. Mar. Biol. Res..

[17] Fernández M, Astorga A, Navarrete SA, Valdovinos C, Marquet PA (2009). Deconstructing latitudinal species richness patterns in the ocean: does larval development hold the clue?. Ecol. Lett..

[18] Lee MR, Riveros M (2012). Latitudinal trenes in the species richness of free-living marine nematodo assemblages from exponed sandy beaches along the coast of Chile (18-42 °S). Mar. Ecol..

[19] Sigwart, J. D. What Species Mean: A User’s Guide to the Units of Biodiversity. 242 pp. (C.R.C. Press, 2018).

[20] Broitman BR, Navarrete SA, Smith F, Gaines SD (2001). Geographic variation of southeastern Pacific intertidal communities. Mar. Ecol. Prog. Ser..

[21] Broitman RB (2011). Geographic variation in diversity of wave exposed rocky intertidal communities along central Chile. Rev. Chil. Hist. Nat..

[22] Rivadeneira MM, Fernández M, Navarrete SA (2002). Latitudinal trends of species diversity in rocky intertidal herbivore assemblages: spatial scale and the relationship between local and regional species richness. Mar. Ecol. Prog. Ser..

[23] Camus PA (2008). Diversidad, distribución y abundancia de especies en ensambles intermareales rocosos. Rev. Biol. Mar. Oceanogr..

[24] Sepúlveda RD, Camus PA, Moreno CA (2016). Diversity of faunal assemblages associated with ribbed mussel beds along the South American coast: relative roles of biogeography and bioengineering. Mar. Ecol..

[25] Jablonski D (2013). Out of the tropics, but how? Fossils, bridge species, and thermal ranges in the dynamics of the marine latitudinal diversity gradient. Proc. Nat. Acad. Sci..

[26] Brattström H, Johanssen A (1983). Ecological and regional zoogeography of the marine benthic fauna of Chile. Sarsia.

[27] Lancellotti DA, Vásquez JA (2000). Zoogeografía de macroinvertebrados bentónicos de la costa de Chile: contribución para la conservación marina. Rev. Chil. Hist. Nat..

[28] Aguilera MA (2005). Cirripedios en la dieta del molusco herbívoro *Chiton granosus* Frembly, 1827 (Mollusca, Placophora) presente en el intermareal rocoso de Iquique, norte de Chile. Invest. Mar..

[29] Aguilera MA, Navarrete SA (2007). Effects of *Chiton granosus* (Frembly, 1827) and other molluscan grazers on algal succession in wave exposed mid-intertidal rocky shores of central Chile. J. Exp. Mar. Biol. Ecol..

[30] Sanhueza AG, Navarrete AH, Opazo LF, Camus PA (2008). Caracterización trófica del placóforo intermareal *Enoplochiton niger* en el norte de Chile: variación ambiental y patrones dietarios a nivel local y región. Rev. Chil. Hist. Nat..

[31] Camus PA, Daroch K, Opazo LF (2008). Potential for omnivory and apparent intraguild predation in rocky intertidal herbivore assemblages from northern Chile. Mar. Ecol. Prog. Ser..

[32] Camus PA, Navarrete AH, Sanhueza AG, Opazo LF (2012). Trophic ecology of the chiton *Acanthopleura echinata* on Chilean rocky shores. Rev. Chil. Hist. Nat..

[33] Camus PA, Arancibia PA, Ávila-Thieme MI (2013). A trophic characterization of intertidal consumers on Chilean rocky shores. Rev. Biol. Mar. Oceanogr..

[34] Eernisse, D. J. Chitons in *Encyclopedia of tidepools and rocky shores* (eds Denny, M. W. & Gaines, S. D.) 127–133 (University of California Press, 2007).

[35] Schwabe E, Försterra G, Häussermann V, Melzer RR, Schrödl M (2006). Chitons (Mollusca: Polyplaophora) from the southern Chilean Comau Fjord, with reinstatement of *Tonicia calbucensis* Plate, 1897. Zootaxa.

[36] Reid DG, Osorio C (2000). The shallow-water marine mollusca of the Estero Elefantes and Laguna San Rafael, southern Chile. Bull. Mus. Nat. Hist. Zool..

[37] Otaíza RD, Santelices B (1985). Vertical distribution of chitons (Mollusca: Polyplacophora) in the rocky intertidal zone of central Chile. J. Exp. Mar. Biol. Ecol..

[38] Aldea C, Valdovinos C (2005). Moluscos del intermareal rocoso del centro-sur de Chile (36°-38°S): Taxonomía y clave de identificación. Gayana.

[39] Araya JF, Araya ME (2015). The shallow-water chitons (Mollusca, Polyplacophora) of Caldera, Region of Atacama, northern Chile. Zoosyst. Evol..

[40] García-Ríos CI, Álvarez-Ruiz M (2007). Comunidades de quitones (Mollusca: Polyplacophora) de la Bahía de La Paz, Baja California Sur, México. Rev. Biol. Trop..

[41] Jörger KM, Meyer R, Wehrtmann IS (2008). Species composition and vertical distribution of chitons (Mollusca: Polyplacophora) in a rocky intertidal zone of the Pacific coast of Costa Rica. J. Mar. Biol. Ass. U.K..

[42] Ibáñez C, Sellanes J, Pardo-Gandarillas MC (2016). Diversidad de poliplacóforos tropicales del sur de la Provincia Panameña. *Lat. Am*. J. Aquat. Res..

[43] Navarrete AH, Lagos NA, Ojeda FP (2014). Latitudinal diversity patterns of Chilean coastal fishes: searching for causal processes. Rev. Chil. Hist. Nat..

[44] Paterson GL, Wilson GD, Cosson N, Lamont PA (1998). Hessler and Jumars (1974) revisited: abyssal polychaete assemblages from the Atlantic and Pacific. Deep Sea Res. Part II.

[45] Clarke A, Lidgard S (2000). Spatial patterns of diversity in the sea: bryozoan species richness in the North Atlantic. J. Anim. Ecol..

[46] Ellingsen KE (2001). Biodiversity of a continental shelf soft-sediment macrobenthos community. Mar. Ecol. Prog. Ser..

[47] Ellingsen KE (2002). Soft-sediment benthic biodiversity on the continental shelf in relation to environmental variability. Mar. Ecol. Prog. Ser..

[48] Ricotta C, Burrascano S (2008). Beta diversity for functional ecology. Preslia.

[49] Baselga A (2010). Partitioning the turnover and nestedness components of beta diversity. Glob. Ecol. Biogeogr..

[50] Figueroa, D. Forcing of physical exchanges in the nearshore Chilean ocean. In *The oceanography and ecology of the nearshore and bays in* Chile (eds Castilla, J. C. & Largier, J. L.) 31–43 (Ediciones Universidad Católica de Chile, 2002).

[51] Rangel TF, Diniz‐Filho JAF, Bini LM (2010). SAM: a comprehensive application for spatial analysis in macroecology. Ecography.

[52] Watters GT (1991). Utilization of a simple morphospace by polyplacophorans and its evolutionary implications. Malacologia.

[53] Montecino, V. *et al*. Bio-physical interactions off western South America (6,E). In *The* Sea (eds Robinson, A. R. & Brink, K. H.) 329–390 (Harvard University Press, 2005).

[54] Vermeij GJ (2012). The evolution of gigantism on temperate seashores. Biol. J. Linn. Soc..

[55] Bullock RC (1988). The genus Chiton in the New World (Polyplacophora: Chitonidae). Veliger.

[56] Kaas, P., Van Belle, R. A. & Stack, H. Monograph of living chitons (Mollusca: Polyplacophora) in *Vol. 6, Suborder Ischnochitonina (concluded); Schizochitonidae & Chitonidae, Additions to Vol. 1–5*. (Koninklijke Brill N.V., 2006).

[57] Schwabe, E. A. Polyplacophora – Chitones (Quitones). In *Fauna marina bentónica de la Patagonia Chilena* (eds Haüssermann, V. & Försterra, G.) 390–424 (Nature in Focus, 2009).

[58] Ibáñez CM (2019). Phylogeny, divergence times, and species delimitation of *Tonicia* (Polyplacophora: Chitonidae) from the Eastern Pacific Ocean. Zool. J. Linnean. Soc..

[59] Colwell RK, Mao CX, Chang J (2004). Interpolating, extrapolating, and comparing incidence‐based species accumulation curves. Ecology.

[60] Koleff P, Gaston KJ, Lennon JJ (2003). Measuring beta diversity for presence-absence data. J. Anim. Ecol..

[61] Condit R (2002). Beta-diversity in tropical forest trees. Science.

[62] Mantel N (1967). The detection of disease clustering and a generalized regression approach. Cancer Res..

[63] Clarke KR (1993). Nonparametric multivariate analyses of changes in community structure. Aust. J. Ecol..

[64] Anderson MJ (2001). A new method for non-parametric multivariate analysis of variance. Austral Ecol..

[65] Hijmans RJ, Guarino L, Cruz M, Rojas E (2001). Computer tools for spatial analysis of plant genetic resources data: 1. DIVA-GIS. Plant Genet. Resour. News..

[66] Hammer, Ø., Harper, D. A. T. & Ryan, P. D. PAST: Paleontological statistics software package for education and data analysis. *Palaeontol. Electron*. **4**, 1–9 (2001).

[67] R Core Team. R. A language and environment for statistical computing. *R Foundation for Statistical Computing, Vienna, Austria*. http://www.R-project.org/ (2018).

[68] Gotelli, N. J. & Entsminger, G. L. EcoSim: Null models software for ecology. Version 5.0. *Acquired Intelligence Inc. & Kesey-Bear*, http://homepages.together.net/~gentsmin/ecosim.htm (2000).

[69] Sigwart JD, Schwabe E (2017). Anatomy of the many feeding types in polyplacophoran molluscs. Invert, Zool..

