# Supplementary material for: Environmental and ecological factors mediate taxonomic composition and body size of polyplacophoran assemblages along the Peruvian Province
Source: Sci Rep. 2019 Nov 4;9:15934. doi: 10.1038/s41598-019-52395-z (PMC6828727; doi:10.1038/s41598-019-52395-z)
Supplement: Supplementary file 1 — Supplementary [file 41598_2019_52395_MOESM1_ESM.docx]

**Environmental and ecological factors mediate taxonomic composition and body size of polyplacophoran assemblages along the Peruvian Province**

Christian M. Ibáñez^1,*^, Melany Waldisperg^2^, Felipe I. Torres^1,3^, Sergio A. Carrasco^4,5^, Javier Sellanes^4,5^, M. Cecilia Pardo-Gandarillas^3,^ Julia D. Sigwart^6^

^1^Departamento de Ecología y Biodiversidad, Facultad de Ciencias de la Vida, Universidad Andres Bello, Santiago, Chile.

^2^Departamento de Ecología, Facultad de Ciencias Biológicas, Pontificia Universidad Católica de Chile, Santiago, Chile.

^3^Departamento de Ciencias Ecológicas, Facultad de Ciencias, Universidad de Chile, Santiago, Chile.

^4^Departamento de Biología Marina, Facultad de Ciencias del Mar, Universidad Católica del Norte, Larrondo 1281, Coquimbo, Chile.

^5^Millennium Nucleus for Ecology and Sustainable Management of Oceanic Islands (ESMOI), Coquimbo, Chile.

^6^Marine Laboratory, Queen’s University Belfast, Portaferry, N. Ireland.

*Corresponding author: C.M. Ibáñez, Departamento de Ecología y Biodiversidad, Facultad de Ciencias de la Vida, Universidad Andres Bello, República 440, Santiago, Chile. Phone: +56-227703890, email: [ibanez.christian@gmail.com](mailto:ibanez.christian@gmail.com), ORCID ID: <https://orcid.org/0000-0002-7390-2617>

**SUPPLEMENTARY MATERIAL**

**Table S1.** Similarity percentage analysis (SIMPER) of relative abundance of species per ecoregion. Values are mean of RA of each species by ecoregion.

| Species | Contribution (%) | Cumulative (%) | | Humboldtian | Central Chile | Araucanian |
| --- | --- | --- | --- | --- | --- | --- |
| \| *Chiton cumingsii* \| \| --- \| \| *Chiton magnificus* \| \| *Chiton granosus* \| \| *Tonicia calbucensis* \| \| *Tonicia fremblyana* \| \| *Enoplochiton niger* \| \| *Tonicia chilensis* \| \| *Chiton barnesii* \| \| *Acanthopleura echinata* \| \| *Chaetopleura peruviana* \| \| *Ischnochiton pusio* \| \| *Callistochiton pulchellus* \| \| *Ischnochiton punctulatissimus* \| \| *Calloplax vivipara* \| \| *Chaetopleura benaventei* \| \| *Plaxiphora aurata* \| \| *Ischnochiton stramineus* \| \| *Tonicia disjunta* \| \| *Tonicia swainsoni* \| \| *Chaetopleura hennahi* \| \| *Gallardoia valdiviensis* \| | 20.2  11.7  10.6  9.5  9.1  7.7  6.3  5.4  4.8  3.8  3.1  1.9  1.5  1.2  1.1  0.46  0.36  0.22  0.22  0.11  0.07 | | 20.20  31.94  42.62  52.21  61.37  69.08  75.42  80.90  85.73  89.59  92.67  94.64  96.17  97.38  98.53  98.99  99.36  99.58  99.81  99.92  100 | 51.9  0  40.4  7.5  31  26.9  0  0.4  14.8  2.5  3.6  7.8  6.1  0.2  0  0.2  0  0  0.7  0.3  0 | 81.1  32.5  18.5  34.7  4.0  7.8  4.44  14.5  13.9  13.3  9.2  0  0  4  0  0.4  0.9  0.8  0  0  0 | 14.2  35.7  45.7  27.3  0.6  0  24.7  0.3  12.2  5.8  0  0  0  0  4.8  1.6  0  0.1  0  0  0.3 |

**Table S2**. Mean and standard deviation of chitons total length (mm) by species and localities.

| **Species** | **Callao** | **Arica** | **Iquique** | **Antofagasta** | **Huasco** | **Coquimbo** | **El Sauce** | **Los Vilos** | **Valparaiso** | **Pichilemu** | **Talcahuano** | **Valdivia** |
| --- | --- | --- | --- | --- | --- | --- | --- | --- | --- | --- | --- | --- |
| *Acanthopleura echinata* | 108.4±12.8 | 79.6±33.7 | 125.4±41.1 | 100.4±22.6 | 115.8±30.9 | 142.5±28.6 |  | 121.3±32.7 | 96.3±56.3 | 75.4±30.2 | 119.2±19.9 |  |
| *Callistochiton pulchellus* |  |  | 11.2±2.0 |  |  |  |  |  |  |  |  |  |
| *Calloplax vivipara* |  |  | 12.5±2.1 |  |  | 10.0±0.0 | 10.2±3.6 | 10.1±4.6 | 14.9±3.7 |  |  |  |
| *Chaetopleura benaventei* |  |  |  |  |  |  |  |  |  |  | 67.1±23.9 | 39.9±21.1 |
| *Chaetopleura hennahi* | 32.7±20.2 |  |  |  |  |  |  |  |  |  |  |  |
| *Chaetopleura peruviana* | 43.0±18.4 | 27.0±5.7 | 23.7±7.5 | 13.7±4.9 | 26.1±13.8 | 21.6±17.4 | 20.4±14.1 | 24.8±15.1 | 25.1±14.1 | 27.4±13.4 | 37.9±16.0 | 43.5±15.3 |
| *Chiton barnesii* |  |  |  | 26.0±4.2 | 14.8±5.4 | 22.9±8.9 | 16.3±8.1 | 26.5±16.3 |  |  |  |  |
| *Chiton cumingsii* | 25.3±9.5 | 32.0±10.3 | 30.7±14.4 | 28.7±11.5 | 24.1±11.7 | 27.1±18.7 | 37.9±12.7 | 36.2±13.8 | 31.2±18.0 | 34.7±10.8 | 34.1±6.8 | 28.3±9.1 |
| *Chiton granosus* | 40.2±13.3 | 36.5±10.2 | 36.1±14.8 | 38.8±16.2 | 48.2±15.0 | 41.4±16.2 | 46.8±8.5 | 51.1±17.6 | 54.1±23.0 | 33.1±16.9 | 43.2±15.7 | 38.2±19.6 |
| *Chiton magnificus* |  |  |  |  | 48.8±28.6 | 85.5±28.5 | 58.4±23.5 | 48.5±31.4 | 30.0±31.0 | 57.9±30.0 | 46.4±14.1 | 46.2±26.6 |
| *Enoplochiton niger* | 50.6±9.4 | 70.4±17.2 | 108.9±37.9 | 73.6±38.3 | 95.1±21.6 | 104.5±35.9 |  |  |  |  |  |  |
| *Gallardoia valdiviensis* |  |  |  |  |  |  |  |  |  |  |  | 20.0 |
| *Ischnochiton punctulatissimus* |  |  | 13.2±3.9 | 18.0 |  |  |  |  |  |  |  |  |
| *Ischnochiton pusio* |  |  |  | 15.7±4.2 | 9.6±2.6 | 14.0±1.7 | 11.0±4.2 | 17.8±2.3 | 12.6±3.5 |  |  |  |
| *Ischnochiton stramineus* |  |  |  |  |  |  | 9.7±3.9 |  |  |  |  |  |
| *Plaxiphora aurata* |  |  |  | 20.0±4.2 |  |  |  |  | 21.3±10.9 |  | 41.7±24.5 | 26.0±7.6 |
| *Tonicia calbucensis* |  | 24.0±9.8 | 27.1±10.2 | 28.2±13.6 | 28.2±9.6 | 40.0±21.0 | 50.0±11.9 | 37.1±15.4 | 36.1±16.4 | 14.7±5.2 | 40.4±7.5 | 42.1±19.8 |
| *Tonicia chilensis* |  |  |  |  | 43.3±1.2 |  |  |  | 61.9±17.3 | 32.4±10.0 | 53.4±25.9 | 40.6±31.9 |
| *Tonicia disjuncta* |  |  |  |  |  |  |  | 55.5±25.1 | 57.5±46.0 |  | 83.6 |  |
| *Tonicia fremblyana* | 27.9±9.6 | 35.1±5.1 | 30.4±8.2 | 36.3±8.4 | 20.8±8.8 | 38.7±8.2 |  |  | 46.1±12.2 | 16.6±3.8 |  |  |
| *Tonicia swainsoni* | 41.0±17.5 |  |  |  |  |  |  |  |  |  |  |  |

**Table S3.** Summary of size ratio null model analyses. Minimum Segment Length (MSL, mm), Standardized Effect Size (SES) and p-value (*P*).

| Locality | Mean MSL | SD MSL | Observed Index | Simulated index | Var. Sim. index | SES | *P* |
| --- | --- | --- | --- | --- | --- | --- | --- |
| Callao | 69.00 | 24.59 | 0.00 | 1.43 | 1.75 | -1.08 | 0.000* |
| Arica | 66.29 | 38.79 | 3.00 | 2.73 | 6.20 | 0.11 | 0.650 |
| Iquique | 70.80 | 67.85 | 2.00 | 1.95 | 3.32 | 0.03 | 0.630 |
| Antofagasta | 59.18 | 47.56 | 1.00 | 1.21 | 1.26 | -0.19 | 0.549 |
| Huasco | 68.97 | 46.74 | 0.70 | 1.24 | 1.36 | -0.47 | 0.419 |
| Coquimbo | 80.52 | 55.29 | 0.00 | 1.50 | 1.90 | -1.09 | 0.000* |
| El Sauce | 48.33 | 32.07 | 0.00 | 1.30 | 1.41 | -1.09 | 0.000* |
| Los Vilos | 78.80 | 53.50 | 4.00 | 1.70 | 2.51 | 1.45 | 0.907 |
| Valparaiso | 87.25 | 57.62 | 1.00 | 1.31 | 1.49 | -0.25 | 0.520 |
| Pichilemu | 64.71 | 39.67 | 1.60 | 2.10 | 3.67 | -0.26 | 0.508 |
| Talcahuano | 88.88 | 34.05 | 0.60 | 1.25 | 1.41 | -0.55 | 0.365 |
| Valdivia | 65.49 | 28.32 | 4.60 | 1.31 | 1.49 | 2.70 | 0.023* |

*significant p-values < 0.05


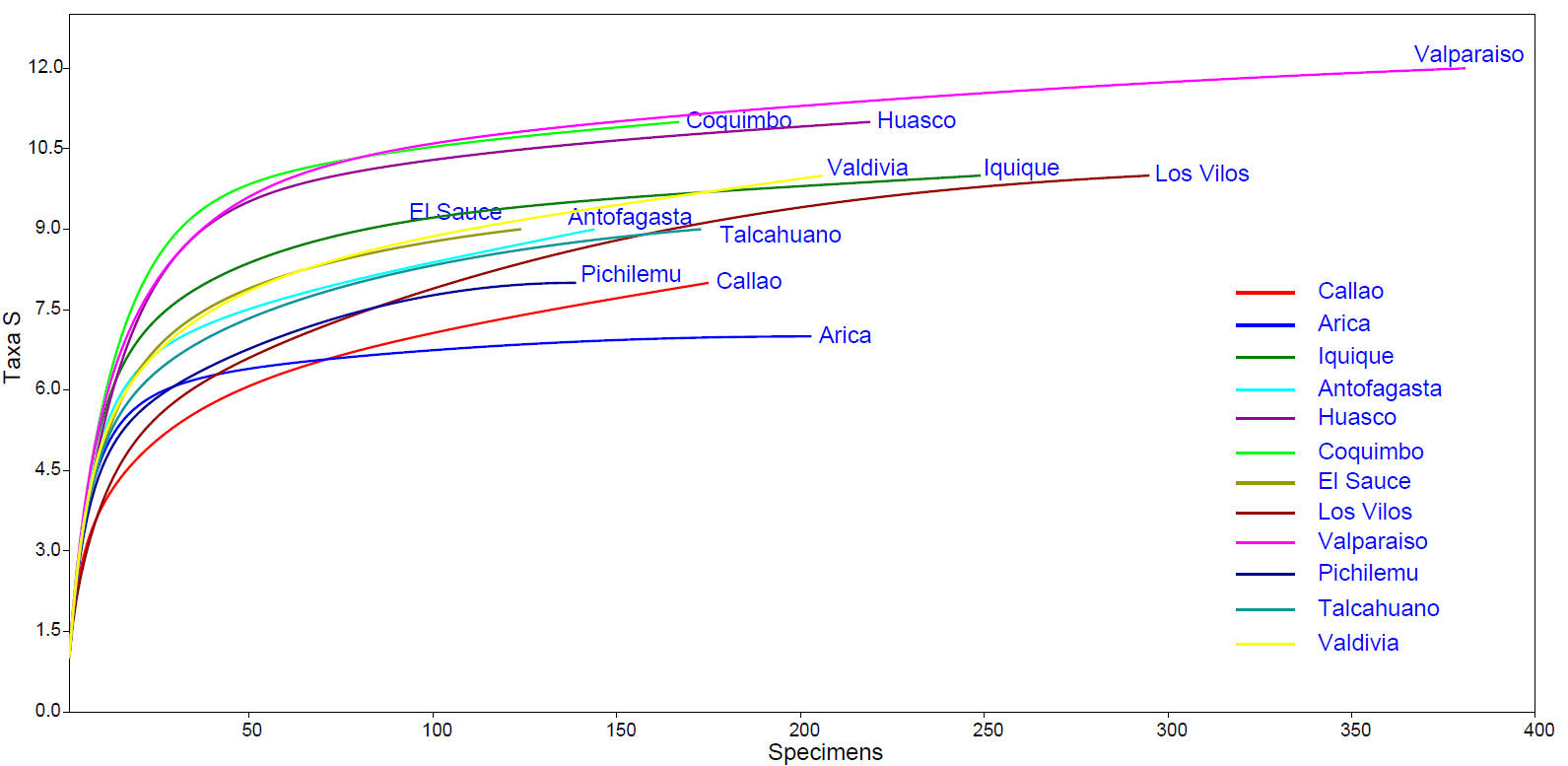


**Figure S1.** Rarefaction curves of the number of chiton species in the Peruvian Province recorded for every locality.
